# Chronic Osteomyelitis of the Jaw. Osteomyelitis

**DOI:** 10.4317/jced.62596

**Published:** 2025-03-01

**Authors:** Carmen López-Carriches, María Victoria Mateos-Moreno, Ricardo Taheri, Juan López-Quiles Martínez, Cristina Madrigal-Martínez-Pereda

**Affiliations:** 1 Associate Professor. Department of Dental Clinic Specialties. School of Dentistry. Universidad Complutense de Madrid. Spain; 2 Doctor of Dental Surgery. DDS. Collaborator. School of Dentistry. Universidad Complutense de Madrid. Spain; 3 Assistant Professor. Department of Dental Clinical Specialties. School of Dentistry. Universidad Complutense de Madrid, Spain

## Abstract

**Background:**

Chronic osteomyelitis of the jaw is an inflammatory reaction of bone tissue of infectious origin that affects the medullary cavity. The main causes of osteomyelitis are odontogenic or traumatic.

**Material and Methods:**

Bibliographic research, the following electronic databases have been searched: Pubmed Medline and the Chochrane Library Plus.

**Results:**

Clinical symptoms are pain, inflammation, suppuration, intraoral or extraoral drainage fistulas. Bone and soft tissues that do not respond favorably to treatment, potentially can lead to bone sequestra. Diagnosis should include a histopathological study throughout a proper biopsy. Identifying the responsible microorganisms is not easy, as the sample can be contaminated by nearby sites. However, a presumptive diagnosis can be made through clinical and radiographic evaluation. Treatment for osteomyelitis involves eliminating the source of infection and necrotic tissue, establishing drainage, restoring blood supply, and controlling the infection with appropriate antimicrobial therapy. Broad-spectrum antibiotics like penicillin or clindamycin are often prescribed initially, but the regimen may be adjusted based on the microbiological findings.

**Conclusions:**

Long-term antibiotic therapy is generally required, ranging from 4 to 6 weeks, depending on the severity and chronicity of the infection.

** Key words:**Chronic Osteomyelitis, antibiotic, mandible, microbiology, surgery.

## Introduction

Chronic osteomyelitis of the jaw is an inflammatory reaction of bone tissue of infectious origin that affects the medullary cavity, Haversian systems, and the adjacent cortical bone.

This inflammatory reaction initially begins as an acute infection. When it lasts more than four weeks, it is called secondary chronic osteomyelitis.

In this review, we will not discuss other forms of osteomyelitis such as osteoradionecrosis, bisphosphonate-related osteonecrosis, Garré’s osteomyelitis, or chronic sclerosing osteomyelitis (1).

## Epidemiology

The global availability of antibiotics, along with advancements in dental and medical care, has reduced the incidence of osteomyelitis. According to Koorbusch *et al*. (2), it occurs more frequently in the jaw, with a wide age range and a higher incidence in men. Haeffs’ *et al*. 10-year study (3) found 62% of cases affected women, with an average age of 53 years.

Sood’s *et al*. retrospective study (4) also found a higher incidence in the lower jaw (55.55%) in comparison with the maxilla.

Fenelon (1) found a slightly higher incidence in women (53.7%) and an average age of 47.5 years.

## Etiopathogenesis

The main causes of osteomyelitis are odontogenic (dental infection) or traumatic (fracture). Patients with osteomyelitis commonly have experienced a traumatic event such as a traumatic tooth extraction, a chronically inflamed carious tooth, a periapical abscess, an infection of adjacent soft tissues, or advanced chronic periodontitis.

Contaminated facial fractures, dental implants, wires, microplates, or mini-screws can also trigger osteomyelitis (2).

In Fenelon’s retrospective study (1), 68% of patients had a history of infection or dental treatment, and 16% had been placed dental implants.

Implant-induced osteomyelitis has a very low incidence, estimated in some studies at only 0.02% (5). This cause responds poorly to treatment and often requires additional surgeries because the surface of implants promotes bacterial adhesion (6). Cases of osteomyelitis associated with dental implants are increasingly being reported in the literature (7). Immediately placed implants, history of diabetes (8), or smoking women have been described as risk factors (9,10).

In many cases, the patient’s medical history may reveal a compromised resistance due to an altered immune status. Some of these risk factors described are the presence of neoplasia, tuberculosis, syphilis, malnutrition, metabolic diseases, immunosuppression, advanced age, alcohol or tobacco abuse, etc.

Many studies have found an increased relation to these risk factors. Koorbusch (2) found that among patients with osteomyelitis, 33.3% had cardiovascular disease, 13% were diabetic, 9.3% were immunocompromised, 7.4% had inflammatory rheumatic disease, and 5.6% were malnourished. Also, half were smokers, 20.4% consumed alcohol, and 7.4% were drug addicts.

In Sood’s study (4), almost 78% of patients had an underlying disease, and 48% used some substance. Moreover, 74% of the diagnosed cases were related to a dental risk factor.

Haeffs (3) also found comorbidities in osteomyelitis patients, particularly cardiovascular disease (52%), tobacco addiction (45%), and psychiatric issues (45%).

The most important factors in the disease’s progression are the virulence of the causative microorganisms, the anatomical characteristics that allow the infection to spread, and the immune response (1).

The primary bacteria associated with this condition is *Staphylococcus aureus*. Although, it is not the only pathogen in jaw osteomyelitis, as the mouth harbors a wide microbiome associated with the teeth and supporting tissues, which can act as pathogens. These include *Streptococcus, Bacteroides*, and other opportunistic bacteria. Impaired vascular perfusion is an important factor in the onset and persistence of the condition. The vascularization of the jaw, in fact, makes it susceptible to osteomyelitis (11).

In Fenelon’s study (1), Streptococcus species were isolated in 40.7% of the samples: *S. constellatus*, *S. intermedius*, and *S. anginosus*, followed by *S. mitis* in 26.2%. Other isolated bacteria were *Actinomyces* (15.1%), *Staphylococcus epidermidis* (7.5%), *Fusobacterium* (3.8%), *Prevotella* (3.8%), *Veillonella* (1.9%), *Parvimonas micra* (1.9%), *Eikenella corrodens* (3.8%), *Klebsiella oxytoca* (1.9%), *Campylobacter rectus* (1.9%), *Staphylococcus capitis* (3.8%), *Escherichia coli* (1.9%), and *Micrococcus luteus* (1.9%).

Haeffs *et al*. (3), found that Streptococcus was isolated in 74% of the samples and Staphylococcus in 43%, showing antibiotic resistance.

## Clinical presentation

Chronic osteomyelitis clinical symptoms are pain, inflammation, suppuration, intraoral or extraoral drainage fistulas. Bone and soft tissues that do not respond favorably to treatment, potentially can lead to bone sequestra (1).

In Koorbusch’s study (3), 74% of patients had inflammation, 71% had pain, 37% had a drainage fistula, and only 3%-6% suffered pathological fractures, sequestra, exposed bone, trismus, and fever occurred.

The patient may also experience anesthesia or paresthesia of the dental nerve (this is a late symptom of the disease process) (3). Regional lymphadenopathy is usually present (1).

## Diagnosis

Diagnosis should include a histopathological study throughout a proper biopsy. Identifying the responsible microorganisms is not easy, as the sample can be contaminated by nearby sites. Sometimes, no pathogens are found in the microbiological culture, and only normal microbiome from the mouth and throat are present (1).

However, a presumptive diagnosis can be made through clinical and radiographic evaluation.

Traditionally, diagnosis by imaging has been performed with a panoramic radiograph, but this method has issues with superimpositions and does not show relevant changes in early stages (12). Therefore, more comprehensive methods such as computed tomography (CT), cone-beam computed tomography (CBCT), single-photon emission computed tomography (SPECT), positron emission tomography (PET), magnetic resonance imaging (MRI), and radionuclide bone scans are advised to be used (13).

CBCT findings in osteomyelitis cases typically show a sclerotic ring with a loss of bone trabeculation and reduction of the alveolar cortical bone, with occasional bone sequestra (14). The local extent of the disease and its relationship with underlying structures are well appreciated with CBCT, and the diagnosis is even more accurate if combined with scintigraphy (15).

SPECT and PET use radiopharmaceuticals that act at the molecular level, providing the exact location of the lesion at an early stage and allowing the treatment response to be evaluated later. Their main drawback is the cost (16,17).

MRI allows for soft tissue evaluation without radiation and is a widely available technique (13,17).

In Fenelon’s retrospective study (1), one or more imaging diagnostic methods were used: CT was the main one (87% of cases), followed by MRI (61%), panoramic radiographs (38.9%), CBCT (11.1%), scintigraphy (7.4%), and PET-CT (7.4%).

Lesions are mainly localized in the molar region and the angle of the jaw. The radiographic appearance of the bone structure varies between patients, although it is usually a poorly defined area of lower bone density with irregular trabecular patterns, in many cases, periosteal reactions can be observed (18).

However, in some patients, the clinical presentation is nonspecific, and radiographic signs are vague, making early diagnosis difficult. Early treatment is crucial for prognosis, depending on early diagnosis. In this regard, bone scintigraphy can be useful, as it is highly sensitive for identifying local bone disease. The detection rate of bone infection ranges from 89% to 100% in studies (11,17). Scintigraphy allows for the detection of osteomyelitis three days after symptom onset. This method also allows to visualize the extent of the lesion, is useful for planning surgical intervention, and helps in obtaining representative biopsy tissues by identifying the region of interest. It also helps to evaluate treatment success, as conventional radiographs continue to show an area of altered bone tissue. With this technique, therapeutic success is associated with a reduction in 99Tc throughout the lesion (18).

Differential diagnosis should be made with several fibro-osseous lesions, such as fibrous dysplasia, which is painless and more frequent in the maxilla (19), or Paget’s disease or osteitis deformans (20). More importantly, it should be distinguished from invasive squamous cell carcinoma of the jaw. Vezcau *et al*. (21) presented a case in which this tumor appeared as chronic osteomyelitis. It should be noted that patients with chronic head and neck inflammation, even in the presence of pain and a fistula, may have malignant neoplasms, especially when antibiotic therapy based provide a limited or no improvement.

According to Hudson *et al*. (11), chronic osteomyelitis can transform to squamous cell carcinoma in 1,5-2% of the cases. Differential diagnosis should be made with sarcoma (22) and osteoblastoma (23).

## Treatment

Treatment for osteomyelitis involves eliminating the source of infection and necrotic tissue, establishing drainage, restoring blood supply, and controlling the infection with appropriate antimicrobial therapy (11).

The approach includes conservative and surgical methods. Conservative treatment is advised in early stages, while surgical treatment is often necessary in advanced or chronic cases, especially when bone sequestra is present (1,2,24).

Conservative treatment includes the use of antibiotics and anti-inflammatory medications. Antibiotic therapy should be used for the specific microorganisms isolated from culture and sensitivity tests. Broad-spectrum antibiotics like penicillin or clindamycin are often prescribed initially, but the regimen may be adjusted based on the microbiological findings. Long-term antibiotic therapy is generally required, ranging from 4 to 6 weeks, depending on the severity and chronicity of the infection (1,22).

Hyperbaric oxygen therapy (HBOT) is another conservative treatment option, especially in cases of osteomyelitis refractory to standard antibiotic therapy. HBOT enhances oxygen supply to the affected tissues, promoting healing by increasing the oxygen concentration in the bone and stimulating osteoclast activity to help remove necrotic bone. Its role is well established in chronic refractory osteomyelitis and osteoradionecrosis (23). According to a review by Lacey *et al*. (24), HBOT is often used as an adjunct therapy and can improve outcomes when combined with surgery and antibiotic therapy.

Surgical intervention is indicated when there is necrotic bone, fistulas, bone sequestra, or when conservative treatment fails. The surgical approach may include sequestrectomy (removal of dead bone), debridement (removal of infected and necrotic tissue), or bone resection (in more severe cases). The goal is to remove all infected tissue and establish a clean area for healing. Bone grafting or reconstruction may be required after extensive resection to restore bone continuity and function (25).

In cases of chronic osteomyelitis, resective surgery combined with local antimicrobial agents, such as antibiotic-impregnated beads or sponges, can help control the infection locally and minimize the need for prolonged systemic antibiotic therapy (26). In some cases, a microvascular free tissue transplant is needed to reconstruct large defects (27).

Fenelon’s study (1) found that 87% of patients required surgery, with 50% needing one or more surgeries. The most common procedures were debridement and sequestrectomy. In some cases, advanced reconstructive surgeries were necessary.

## Postoperative care and follow-up

Postoperative care includes long-term antibiotics, regular imaging to monitor the healing process, and management of any potential complications. Proper oral hygiene and elimination of the primary infection source are crucial to prevent recurrence.

In cases involving dental implants, the implants are often removed, especially if they are the cause of infection or if the surrounding bone is affected. If the patient desires to replace the removed implants, they can be considered after a complete healing and the resolution of the infection.

## Prognosis

The prognosis of chronic osteomyelitis of the jaw depends on several factors, including the promptness of diagnosis, the virulence of the pathogen, the patient’s immune status, and the effectiveness of treatment. Early intervention generally leads to better outcomes, while delayed diagnosis or inappropriate treatment can lead to chronic infection, bone loss, and the need for more extensive surgery.

Studies suggest that when treated adequately, patients can achieve full recovery. However, long-term follow-up is necessary to monitor for recurrence. In some cases, chronic osteomyelitis can lead to complications such as pathological fractures, osteonecrosis, or the spread of infection to adjacent structures, which can severely impact the patient’s quality of life (1,12.

## Conclusions

Chronic osteomyelitis of the jaw is a serious condition that requires a multidisciplinary approach involving maxillofacial surgeons, infectious disease specialists, and radiologists. The timing of the diagnosis and appropriate treatment are crucial for preventing severe complications and ensuring optimal outcomes. Advanced imaging techniques, combined with microbiological analysis, are essential for accurate diagnosis, while treatment should be customized based on the specific needs of the patient. Conservative therapies, including antibiotics and HBOT, are useful in early stages, but surgery is often necessary in more advanced cases.

Further research is needed to better understand the factors contributing to the disease’s pathogenesis and to develop more effective treatment strategies for this potentially debilitating condition.

## Data Availability

The datasets used and/or analyzed during the current study are available from the corresponding author.
